# A Complex Interplay between Nitric Oxide, Quorum Sensing, and the Unique Secondary Metabolite Tundrenone Constitutes the Hypoxia Response in *Methylobacter*

**DOI:** 10.1128/mSystems.00770-19

**Published:** 2020-01-21

**Authors:** Zheng Yu, Mitchell Pesesky, Lei Zhang, Jing Huang, Mari Winkler, Ludmila Chistoserdova

**Affiliations:** a Department of Chemical Engineering, University of Washington, Seattle, Washington, USA; b Department of Civil and Environmental Engineering, University of Washington, Seattle, Washington, USA; c Department of Microbiology, School of Basic Medical Science, Central South University, Changsha, Hunan, China; Michigan State University

**Keywords:** hypoxia, *Methylobacter tundripaludum*, nitric oxide, hybrid cluster protein, quorum sensing, tundrenone

## Abstract

*Methylobacter* species, members of the *Methylococcales*, have recently emerged as some of the globally widespread, cosmopolitan species that play a key role in the environmental consumption of methane across gradients of dioxygen tensions. In this work, we approached the question of how *Methylobacter* copes with hypoxia, via laboratory manipulation. Through comparative transcriptomics of cultures grown under high dioxygen partial pressure versus cultures exposed to hypoxia, we identified a gene cluster encoding a hybrid cluster protein along with sensing and regulatory functions. Through mutant analysis, we demonstrated that this gene cluster is involved in the hypoxia stress response. Through additional transcriptomic analyses, we uncovered a complex interconnection between the NO-mediated stress response, quorum sensing, the secondary metabolite tundrenone, and methanol dehydrogenase functions. This novel and complex hypoxia stress response system is so far unique to *Methylobacter* species, and it may play a role in the environmental fitness of these organisms and in their cosmopolitan environmental distribution.

**IMPORTANCE** Here, we describe a novel and complex hypoxia response system in a methanotrophic bacterium that involves modules of central carbon metabolism, denitrification, quorum sensing, and a secondary metabolite, tundrenone. This intricate stress response system, so far unique to *Methylobacter* species, may be responsible for the persistence and activity of these species across gradients of dioxygen tensions and for the cosmopolitan distribution of these organisms in freshwater and soil environments in the Northern Hemisphere, including the fast-melting permafrosts.

## Abstract (precis)

Here, we describe a novel and complex hypoxia response system in a methanotrophic bacterium that involves modules of central carbon metabolism, denitrification, quorum sensing, and a secondary metabolite, tundrenone. This intricate stress response system, so far unique to *Methylobacter* species, may be responsible for the persistence and activity of these species across gradients of dioxygen tensions and for the cosmopolitan distribution of these organisms in freshwater and soil environments in the Northern Hemisphere, including the fast-melting permafrosts.

## INTRODUCTION

*Methylobacter* species, members of the *Methylococcales* that are gammaproteobacterial aerobic methanotrophs, have recently emerged as some of the globally widespread, cosmopolitan species, appearing to play a key role in the consumption of methane in a variety of environments and across gradients of dioxygen tensions (1–3). While multiple species of both gamma- and alphaproteobacterial methanotrophs are detectable in environmental samples using molecular techniques (1–4), and many are also available in pure cultures (5, 6), *Methylobacter* appears to be the dominant and the most active species in freshwater and soil environments of the Northern Hemisphere (1–4). Some of the environments hosting *Methylobacter*, such as permafrosts, represent vast areas on this planet, which are also subject to rapid environmental change as part of the global warming effect, manifested by increased methane release resulting from ice melt (7, 8). Importantly, *Methylobacter* activities have been correlated with methane consumption not only in oxic but also in hypoxic and even anoxic niches (9–12), even though the mechanisms for such activity remain poorly understood, given the essential role of dioxygen in methane oxidation catalyzed by methane monooxygenase (MMO). The observations on the dominant nature of *Methylobacter* have also been supported by results from microcosm incubation experiments in which *Methylobacter* performed as the most competitive species, especially under low dioxygen tensions (13, 14).

In addition to their proficiency in oxidizing methane, under a variety of conditions, *Methylobacter* species have also been implicated in shuttling carbon from methane through a bacterial food chain, supporting diverse communities and linking methane consumption to other important biogeochemical processes (15). Methanol-utilizing *Methylophilaceae* have been noted as some of the most persistent partners of *Methylobacter* in both natural environments (3) and laboratory simulations (13, 14). Experiments involving synthetic community manipulations have suggested that the methanol-consuming partner species may be capable of modifying carbon metabolism in *Methylobacter*, by affecting the expression of alternative methanol dehydrogenase (MDH) enzymes, and thus potentially modulating the supply of methanol to the satellite populations of *Methylophilaceae* (16). Other products that may be released by *Methylobacter* have been hypothesized to be organic acids and polymeric substances, which would support populations of nonmethylotrophic heterotrophs (15, 17).

What makes *Methylobacter* such an environmentally successful species? It is unlikely that these are its growth characteristics, as in the laboratory, *Methylobacter* does not perform robustly and is easily outcompeted by other species such as *Methylomonas* (18, 19). Toward the potential for methane oxidation in niches depleted of dioxygen, the denitrification capability has been proposed to be one metabolic means for coping with hypoxia (20, 21). However, transcriptomics studies applied to natural samples with highly active *Methylobacter* populations failed to account for the transcription of the denitrification pathway (2).

In this study, we approached the question of how *Methylobacter* copes with hypoxia that it encounters in natural environmental niches, via laboratory manipulation. We first carried out comparative transcriptomics with cultures grown under high dioxygen partial pressure and cultures starved for dioxygen, identifying genes showing differential transcription. We then mutated several genes of interest and investigated mutant phenotypes. We followed up with additional transcriptomic analyses comparing gene expression in mutant versus wild-type backgrounds and comparing expression patterns between cultures provided with ammonium and those provided with nitrate as nitrogen sources. We uncovered an unexpected connection between the hypoxia stress response and quorum sensing (QS) functions, which, in turn, regulate the expression of a gene cluster responsible for the synthesis of a secondary metabolite, tundrenone (Tun). We also obtained evidence for nitric oxide (NO), generated in the denitrification pathway, playing a role in the stress response, likely as a signaling molecule. We further uncovered that the differential expression of genes for the alternative MDH enzymes was also part of the hypoxia response.

## RESULTS

### Comparative transcriptomics point to the role of Hcp/Hcr in the hypoxia response by *Methylobacter*.

We exposed cultures of Methylobacter tundripaludum strain 31/32 to hypoxia, sampling cells 24 and 48 h after the last addition of O_2_, and compared the transcriptomes of the dioxygen-starved cultures to the transcriptomes of the oxygenated cultures (transcriptomics experiment 1) (see Materials and Methods). Two different experiments (experiments A and B) were run in two replicates each, all involving synthetic community mixtures (see Materials and Methods) (17). For the purposes of this study, we focused solely on the transcriptomes of M. tundripaludum 31/32.

**FIG 1:**
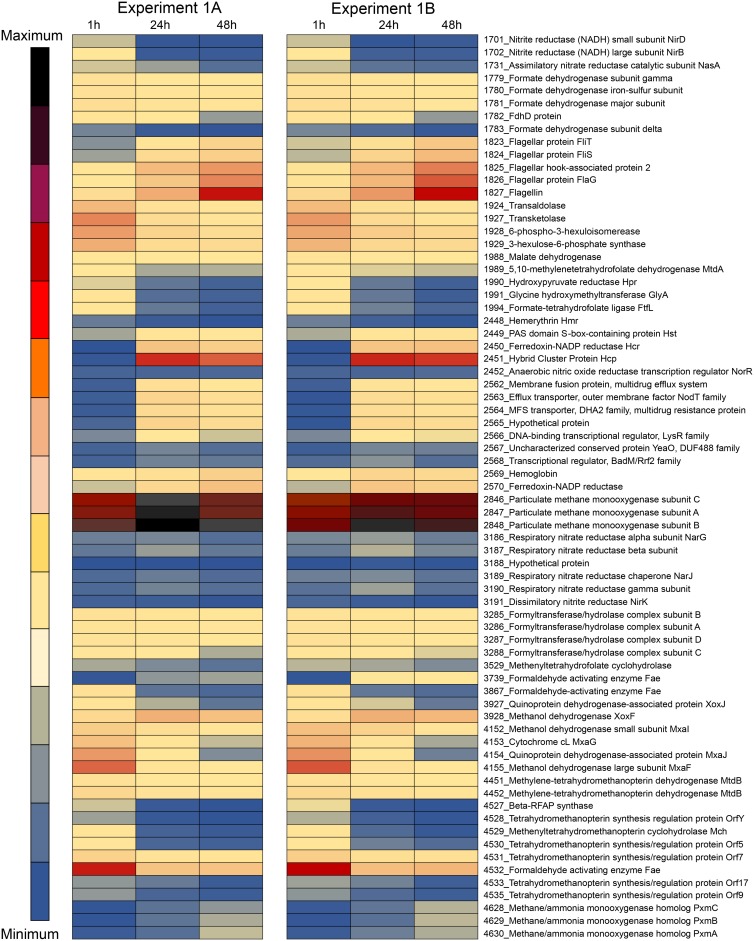
Heat map depicting differential expression of select genes in M. tundripaludum 31/32 under oxic (1 h) and increasingly hypoxic (24 and 48 h) conditions (experiments 1A and B). A single replicate for each experiment is shown. Complete transcriptomics data, including additional replicates, are presented in Tables S1 and S2 in the supplemental material. MFS, major facilitator superfamily.

**FIG 2:**
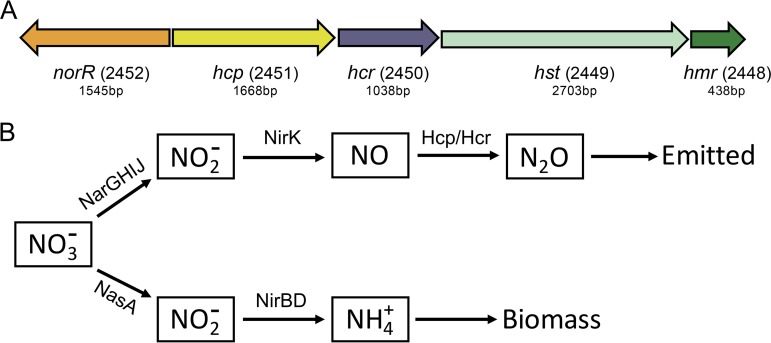
(A) Schematic depicting a gene cluster encoding hybrid cluster protein (Hcp) and a cognate flavodoxin reductase (Hcr). NorR is annotated as an anaerobic NO reductase transcriptional regulator. Hst is a proposed name for the protein that appears to be involved in sensing O_2_ and/or NO (hypoxia signal transduction). Hmr is a hemerythrin. Gene numbers (in parentheses) as well as the respective gene sizes are shown. (B) Schematic depicting the dissimilatory and assimilatory denitrification pathways, with Hcp/Hcr serving as a NO reductase. No genes are recognized in the genome that would encode an alternative NO reductase or an N_2_O reductase.

Dramatic changes in transcription patterns were determined for cultures exposed to hypoxia, compared to the oxygenated cultures (Fig. 1; see also Tables S1 and S2 in the supplemental material). In general, among the two independent experiments, the transcription of over 500 genes was downregulated more than 10-fold, while the transcription of about 150 genes was upregulated more than 10-fold, in response to hypoxia. Between the two experiments, the trends observed were very similar (Fig. 1; Tables S1 and S2). The expression of two genes was dramatically upregulated (up to 4,000-fold) in response to hypoxia, encoding a hybrid cluster protein (Hcp) (gene 2451) and a cognate flavodoxin reductase (Hcr) (gene 2450). A closer look uncovered a long history of misannotation of the gene for Hcp. In most genomes, it is annotated as a hydroxylamine reductase, with this persistent misannotation stemming from the previously reported observation of a secondary, minor side activity of the enzyme (22). In a later study, the respective enzyme was identified as a high-affinity NO reductase (23). Additional studies implicated this protein in nitrosative and oxidative stress responses (24, 25). While the *hcp*-*hcr* genes were previously proposed to be involved in the denitrification pathway in a methanotroph (a *Crenothrix* species [26]), in the experiment presented here, genes for other respiratory and assimilatory nitrate reduction functions were expressed at low levels, and they did not show differential expression in response to the onset of hypoxia (Fig. 1 and Fig. 2B; Tables S1 and S2).

10.1128/mSystems.00770-19.5 TABLE S1 Normalized transcript counts for the wild-type transition from oxic to hypoxic conditions (experiment 1A). Download Table S1, XLSX file, 0.4 MB. Copyright © 2020 Yu et al. 2020 Yu et al. This content is distributed under the terms of the Creative Commons Attribution 4.0 International license.

10.1128/mSystems.00770-19.6 TABLE S2 Normalized transcript counts for the wild-type transition from oxic to hypoxic conditions (experiment 1B). Download Table S2, XLSX file, 0.4 MB. Copyright © 2020 Yu et al. 2020 Yu et al. This content is distributed under the terms of the Creative Commons Attribution 4.0 International license.

One of the most highly downregulated gene clusters in response to hypoxia was the one for the calcium-dependent MDH, the *mxa* cluster (genes 4146 to 4157) (Fig. 1; Tables S1 and S2). Conversely, the gene for an alternative MDH, the lanthanide-dependent XoxF enzyme (gene 3928), was upregulated. A pattern of reversed transcriptional regulation of the alternative MDH enzymes in response to hypoxia has been noted previously (19). As for other methylotrophy functions, the transcription of genes for MMO (genes 2846 to 2848) was slightly upregulated, while the transcription of most of the genes for the assimilatory ribulose monophosphate and the (partial) serine cycles (27, 28) as well as of genes for formaldehyde and formate oxidation was downregulated (up to 45-fold) (Fig. 1; Tables S1 and S2). Overall, the transcription of genes for many essential cellular functions, including ribosomal proteins, was decreased, likely indicating a switch to the oxygen starvation mode.

### Mutants in the *hcp-hcr* gene cluster, key denitrification genes, demonstrate altered hypoxia and oxidative and nitrosative stress responses.

We focused on the cluster of genes whose transcription was most highly induced by hypoxia, *hcp* and *hcr.* Additional genes that were part of the cluster encoded a transcriptional regulator annotated as NorR (anaerobic NO reductase transcriptional regulator), a signal transduction protein possessing PAS, EAL, and GGDEF domains (here named Hst, for hypoxia signal transfer), and a hemerythrin (Hmr), a small diiron protein for the transfer and storage of dioxygen (Fig. 2A). While the Hcp/Hcr pair has been previously demonstrated to act as a high-affinity NO reductase (23), the expression pattern for *hcp*-*hcr*, as reported here, showed a poor correlation with the expression patterns for other genes in the NO-producing denitrification pathway (Fig. 2B), potentially suggesting an additional function for Hcp/Hcr. We hypothesized that this additional function may be in the stress response, as previously reported for other organisms (24, 25). To test our hypothesis, we constructed knockout mutants for each of the genes in the *hcp* gene cluster. To directly test the potential of nitrate in serving as an alternative electron acceptor to allow *Methylobacter* to cope with hypoxia, we mutated the major subunit of the respiratory nitrate reductase (NR) (NarG; gene 3186). As a control, we also mutated the assimilatory NR (NasA; gene 1731). To select for recombinants, we used both nitrate-supplemented (nitrate minimal salts [NMS]) and ammonium-supplemented (ammonium mineral salts [AMS]) selective media. We noted that in each case, colonies appeared only on the AMS and not on the NMS plates, likely suggesting that all the mutants had decreased survival rates when nitrate served as the nitrogen source.

When inoculated into liquid media, all the mutants were able to grow with both nitrate and ammonium as sources of nitrogen, with one exception. The mutant in NasA did not show any appreciable growth in nitrate-supplemented medium, while the remaining mutants showed decreased growth compared to ammonium-supplemented medium and to wild-type controls (Fig. S1). These results suggested that all the functions encoded by the *hcp-hcr* gene cluster and both the assimilatory and the dissimilatory denitrification pathways must be involved in the hypoxia stress response by *Methylobacter*.

10.1128/mSystems.00770-19.1 FIG S1 Growth in NMS and AMS media under high-oxygen (A) and low-oxygen (B) conditions. For high-oxygen conditions, cells were grown under a methane-air (25%:75%, vol/vol) atmosphere, which was replenished once daily. After 35 h, cultures were diluted to an OD_600_ of 0.1 in fresh medium and grown under the same conditions. For low-oxygen conditions, cells were grown under a methane-air-dinitrogen (25%:5%:70%, vol/vol) atmosphere, which was replenished once daily. After 8 days, cultures were diluted to an OD_600_ of 0.1 in fresh medium and grown under the same conditions. Download FIG S1, TIF file, 1.9 MB. Copyright © 2020 Yu et al. 2020 Yu et al. This content is distributed under the terms of the Creative Commons Attribution 4.0 International license.

We tested the survival rate under hypoxic conditions by incubating cells for 40 days without replenishing dioxygen in the headspace atmosphere, monitoring the fate of cell populations every 10 days using LIVE/DEAD cell stain in medium supplemented with either nitrate or ammonium. Over 40 days, approximately 65% of the initial wild-type cell population died out in both types of media, presumably from dioxygen starvation (Fig. S2A). All the mutants demonstrated reduced survival rates compared to the wild type, with the NasA mutant demonstrating a dramatic death rate in nitrate medium while surviving quite well in ammonium medium. Notably, cultures starved for dioxygen do not show significant growth, as reported previously (17, 18). Thus, nitrogen availability should not be essential for building biomass. This suggested that the phenotype of the NasA mutant might be reflective of a specific stress response. The pattern of rapid dying out was further tested in a short-term experiment, over 12 h, carried out with the entire group of strains. In this experiment, only the NasA mutant revealed a significant decline in live cells and only in nitrate-supplemented medium (Fig. S2B). The most parsimonious explanation for such a phenotype would be the redirection of most of the nitrate into the dissimilatory pathway, via respiratory NR (NarGHIJ) and NO-producing nitrite reductase (NirK), resulting in a surge in toxic NO (Fig. 2B).

10.1128/mSystems.00770-19.2 FIG S2 Dead/live cell counts over long-term (A) and short-term (B) incubations without replenishing dioxygen in the headspace. Live/dead cell ratios were determined every 10 days and 4 h for panels A and B, respectively. Download FIG S2, TIF file, 0.7 MB. Copyright © 2020 Yu et al. 2020 Yu et al. This content is distributed under the terms of the Creative Commons Attribution 4.0 International license.

We further employed several stressors that are typically used to test for tolerance to oxidative and nitrosative stresses. When challenged with H_2_O_2_, all the mutants ceased growth, and the number of live cells steadily decreased over the course of the experiment (Fig. S3A), in contrast to the nonstressed cultures (Fig. S3D). The NasA mutant was one exception, and it revealed a steep decline in live-cell numbers when incubated in nitrate medium, even in cultures with no added stressor, further suggesting that a stressor, likely NO, was produced by this mutant. While wild-type cells showed a diminished growth rate under H_2_O_2_ stress, cell death was not observed over the course of the experiment (Fig. S3A). Very similar results were obtained with an alternative oxidative stress agent, paraquat (Fig. S3B).

10.1128/mSystems.00770-19.3 FIG S3 Growth (top) and live/dead cell ratios (bottom) over a 12-h challenge with H_2_O_2_ (A), paraquat (B), and DETA-NO (C) and without any stressors (control) (D). Download FIG S3, TIF file, 2.3 MB. Copyright © 2020 Yu et al. 2020 Yu et al. This content is distributed under the terms of the Creative Commons Attribution 4.0 International license.

As a nitrosative stress agent, we employed a diethylenetriamine (DETA)-NO adduct. When tested under oxic conditions, most of the mutants were able to grow at rates similar to wild-type strain rates with both nitrate and ammonium. The NasA mutant did not grow in nitrate medium, as expected (Fig. S3C). Two other exceptions were the mutants in Hcp and Hcr, showing severely declining growth. This phenotype was consistent with the Hcp/Hcr pair possessing NO reductase activity, with the mutants thus being subject to NO toxicity (Fig. S3C).

### NO and N_2_O measurements support the role of NO in the stress response and the role of Hcp/Hcr in NO reduction.

**FIG 3:**
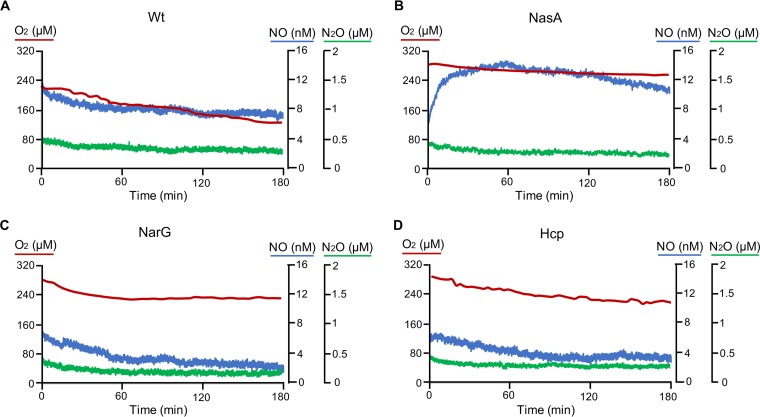
Instantaneous measurements of dissolved O_2_, NO, and N_2_O in select mutant and wild-type (Wt) cells incubated in nitrate-containing medium. The NasA mutant shows immediate accumulation of NO. Long-term kinetics are shown for the Hcp mutant and the wild type in Fig. S4 in the supplemental material.

We directly measured the dynamics of instantaneous concentrations of dissolved NO, N_2_O, and O_2_ in select strain cultures, using microsensors, after the growth medium containing ammonium as the nitrogen source (AMS) was replaced with NMS medium. Upon exposure to nitrate, the NasA mutant immediately accumulated NO. The peak of NO was observed as early as 1 h after exposure to nitrate, while oxygen consumption was very slow (Fig. 3B), corroborating the observations of rapid cell death in the presence of nitrate (Fig. S1 and S2). No short-term NO accumulation was observed when NarG and Hcp mutants or the wild-type strain was exposed to nitrate (Fig. 3A, C, and D). The NarG mutant showed brief dioxygen consumption followed by the loss of metabolic activity. This phenotype is not easy to interpret, but it would be consistent with the involvement of NO in the stress response. Neither NO nor N_2_O was produced by this mutant (Fig. 3C). In the case of the Hcp mutant and the wild-type strain, NO accumulated only after a long lag time (>10 h) (Fig. S4A and B). A peak of N_2_O was measured only in the wild-type strain, with this peak coinciding with the NO peak (Fig. S4A). Notably, NO and N_2_O production by the wild-type strain was observed only after the onset of hypoxia, while the Hcp mutant accumulated NO under oxic conditions (Fig. S4B), suggesting that the denitrification pathway was deregulated in this mutant. The lack of N_2_O production by this mutant further supported the role of Hcp in NO reduction.

10.1128/mSystems.00770-19.4 FIG S4 Instantaneous measurements of dissolved O_2_, NO, and N_2_O in wild-type cells versus Hcp mutant cells over long-term incubation in nitrate-containing medium. (A) Simultaneous NO and N_2_O accumulation by wild-type cells takes place under hypoxic conditions. (B) The Hcp mutant accumulates NO but not N_2_O under oxic conditions. No NO or N_2_O was accumulated by the NarG or NasA mutant in similar experiments under similar conditions (not shown). Download FIG S4, TIF file, 1.1 MB. Copyright © 2020 Yu et al. 2020 Yu et al. This content is distributed under the terms of the Creative Commons Attribution 4.0 International license.

### Lack of Hcp alters the transcription pattern and reveals connections between the hypoxia stress response, quorum sensing, and the secondary metabolite tundrenone.

**FIG 4:**
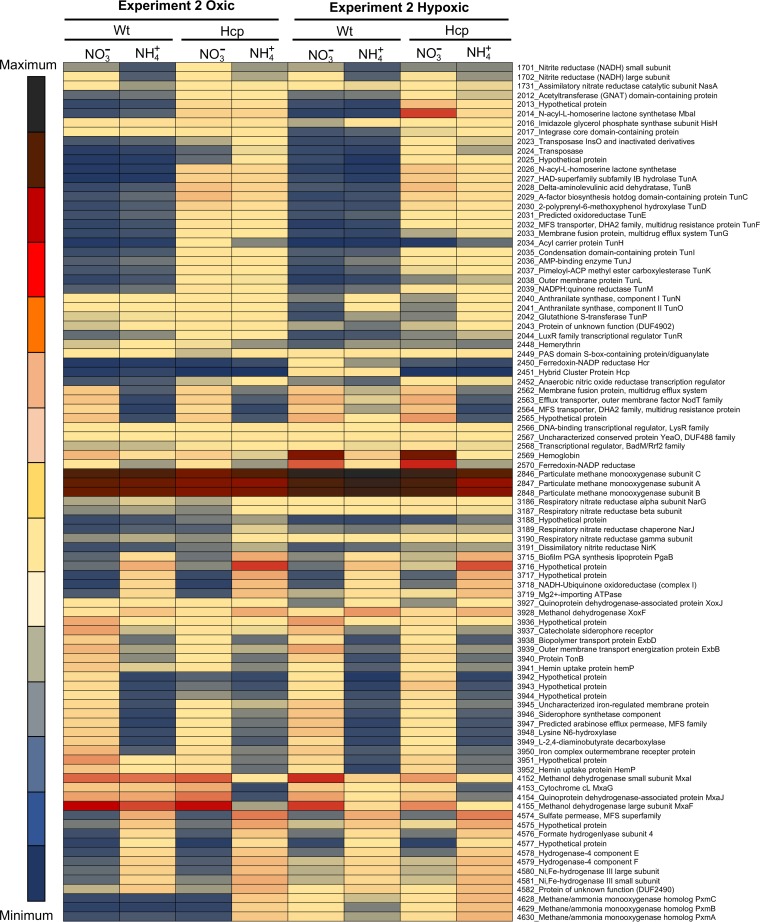
Heat map depicting differential expression of select genes in the Hcp mutant in comparison with wild-type M. tundripaludum 31/32 under four different conditions (experiment 2). A single replicate for each strain/condition is shown. Complete transcriptomics data, including additional replicates, are presented in Table S3 in the supplemental material. GNAT, general control nonrepressed-related *N*-acetyltransferase; HAD, haloacid dehydrogenase; ACP, acyl carrier protein; PGA, poly-beta-1,6-acetyl-d-glucosamine.

**FIG 5:**
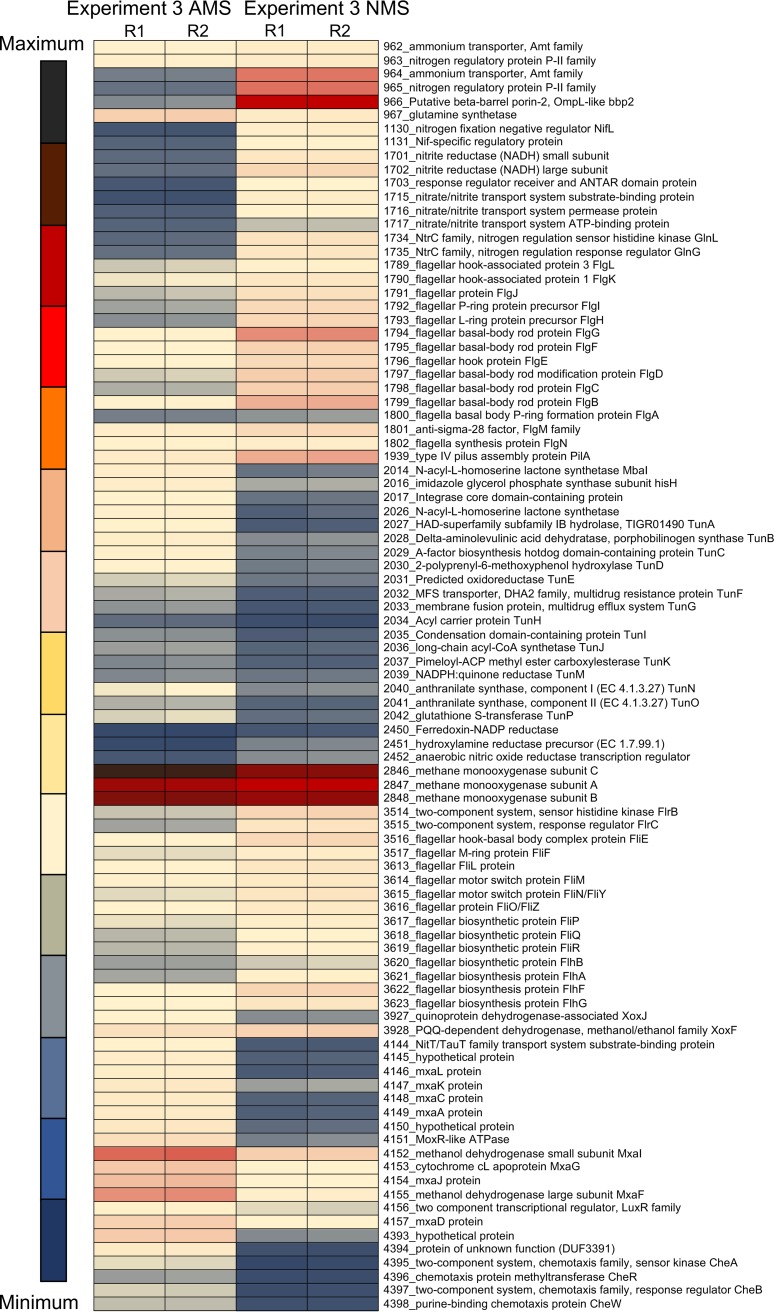
Heat map depicting differential expression of select genes in the NasA mutant challenged with nitrate (experiment 3). R1 and R2 are data from biological replicates. Complete transcriptomics data are presented in Table S4 in the supplemental material. PQQ, pyrroloquinoline quinone.

With transcriptomics experiment 2, we approached the following questions: does the mutation in Hcp cause a transcriptional response, and is this response hypoxia specific? RNA was isolated from cells exponentially growing under oxygenated conditions with nitrate or with ammonium and from cells subjected to dioxygen starvation for 24 h (see Materials and Methods). A total of 16 RNA samples were sequenced, representing the wild type and the Hcp mutant incubated under the four conditions, in two replicates each (Table S3). When the transcriptomes of the Hcp mutant and the wild type were compared, under either condition, the most dramatic response to the lack of Hcp was manifested by the recently characterized gene cluster encoding quorum sensing (QS) functions and the novel secondary metabolite tundrenone (Tun) (29) (Fig. 4). The transcription of a total of 27 genes in the cluster was upregulated, including key genes in Tun biosynthesis. The gene for an *N*-3-hydroxydecanoyl-l-homoserine lactone (HSL) synthase, *mbaI*, showed the most dramatic response, which was most pronounced in cells subjected to hypoxia in nitrate medium, >1,000-fold upregulation. In comparison, the transcription of *mbaI* was >100-fold upregulated in cells subjected to hypoxia in ammonium medium and approximately 50-fold upregulated in cells from the oxygenated cultures (Fig. 5). It has been noted previously that in the MbaI mutant not producing native HSL, when exogenous HSL was added, some of the most downregulated genes were the genes for Hcp and Hcr (29). Thus, it appears that the Hcp/Hcr and the QS/Tun systems are inversely regulated and that they potentially regulate each other. It appears that Hcp/Hcr or the products controlled by this enzyme pair (potentially NO) control the transcription of the QS system and, thus, the production of Tun and that a product positively regulated by the QS system, potentially Tun, controls transcription from *hcp*-*hcr*. Other notable alterations in transcription included the significantly reduced expression of the *mxa* operon in the mutant when grown with ammonium but not nitrate. The *mxa* gene cluster was also downregulated in wild-type cells in response to hypoxia, as has been noted previously (19), but to a much lesser degree than in the Hcp mutant (Fig. 5). This further suggested that an intermediate in the denitrification pathway, likely NO, must be part of the regulatory cascade controlling the expression of the *mxa* gene operon. The levels of NO should be elevated in the Hcp mutant grown with nitrate, thus positively correlating with *mxa* expression. The transcription of the *nar* operon was slightly (∼2-fold) elevated in this mutant (Fig. 4).

10.1128/mSystems.00770-19.7 TABLE S3 Normalized transcript counts for the Hcp mutant versus the wild type (experiment 2). Download Table S3, XLSX file, 1.0 MB. Copyright © 2020 Yu et al. 2020 Yu et al. This content is distributed under the terms of the Creative Commons Attribution 4.0 International license.

10.1128/mSystems.00770-19.8 TABLE S4 Normalized transcript counts for the NasA mutant exposed to nitrate versus ammonium (experiment 3). Download Table S4, XLSX file, 0.4 MB. Copyright © 2020 Yu et al. 2020 Yu et al. This content is distributed under the terms of the Creative Commons Attribution 4.0 International license.

### NO is involved in transcriptional regulation of quorum sensing, tundrenone synthesis, and methanol oxidation.

With transcriptomics experiment 3, we tested whether NO may be directly involved in gene expression regulation and which, if any, functions would be affected by the elevated levels of NO. We employed the NasA mutant, which revealed the most dramatic phenotype for growth with nitrate, and it emitted NO (Fig. 3B). Samples for RNA extraction were taken after 1 h of exposure to nitrate, based on the evidence for immediate NO release by the mutant (Fig. 3B), and these nitrate (NO)-challenged samples were compared to samples incubated with ammonium under fully oxygenated conditions. Overall, the expression of many genes, including most of the methylotrophy genes, was downregulated (3- to 5-fold) after exposure to nitrate, in agreement with the toxic nature of NO. However, some genes showed upregulation. Among these were genes involved in nitrate metabolism functions, such as ammonium transporters, nitrogen regulatory proteins, nitrate/nitrite transporters, as well as the assimilatory nitrite reductase (Fig. 5; Table S4). In addition, the transcription of genes encoding flagella as well as type IV pilus assembly functions was upregulated. Remarkably, some of the regulated gene clusters included those for QS/Tun, Hcp/Hcr, Mxa, and Xox functions. Of these, the transcription of genes encoding QS/Tun as well as Mxa functions showed rather significant (up to 20-fold) downregulation, while the transcription of genes encoding Hcp/Hcr and XoxF was slightly upregulated, once again suggesting an inverse regulation of QS/Tun/Mxa versus Hcp/Hcr/XoxF genes. These results suggested that the transcriptional response to NO must be dose dependent and that NO can serve as either a stimulator or a toxic agent, depending on its intracellular concentration.

### Quorum sensing and tundrenone are involved in the hypoxia response.

**FIG 6:**
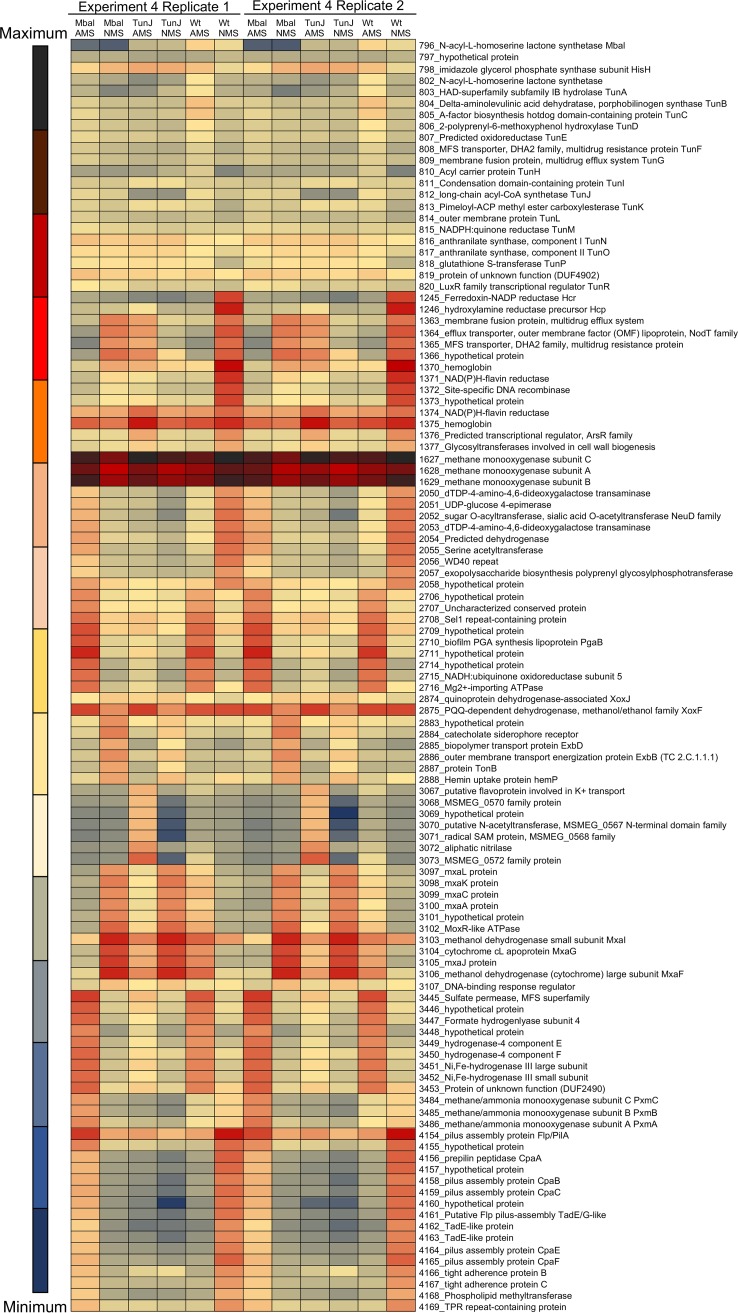
Heat map depicting differential expression of select genes in the MbaI and TunJ mutants in comparison with M. tundripaludum 21/22, grown with either ammonium or nitrate (experiment 4). Complete transcriptomics data are presented in Table S5 in the supplemental material. SAM, *S*-adenosylmethionine.

Transcriptomics experiment 4 was designed to further decipher the potentially interlinked roles of Hcp/Hcr and QS/Tun in the hypoxia response. This experiment was carried out with the previously described mutants in QS (MbaI) (29) and Tun biosynthesis (TunJ) (30). The former mutant does not produce the signal molecule HSL, which induces the transcription of the genes essential for the biosynthesis of Tun. Thus, this mutant produces neither HSL nor Tun under the conditions used (29). The latter mutant does not produce Tun, while it still produces HSL (30). Note that a different but closely related M. tundripaludum strain, 21/22, was employed in these experiments, due to the availability of validated mutants (29, 30). The mutants and the wild type were grown in either nitrate (NMS)- or ammonium (AMS)-containing medium for 48 h, at which stage cell densities sufficient for the production of HSL were reached (29, 30), and at the same time, dioxygen in the cultures was sufficiently depleted to cause hypoxia (13, 14). Remarkably, neither MbaI nor TunJ mutants produced appreciable amounts of *hcp*-*hcr* transcripts with either nitrate or ammonium, revealing phenotypes not responsive to hypoxia. Likewise, the transcriptions of both the *mxa* gene cluster and *xoxF* were deregulated, with the mutants not responding to hypoxia (Fig. 6; Table S5). While MbaI and TunJ mutants showed similar transcription patterns for some functions, they demonstrated different patterns for others, suggesting that HSL and Tun may have overlapping but distinct functions in transcriptional regulation. For example, it appears that in the absence of Tun, the expression levels of the pilus function genes (genes 4154 to 4169), exopolysaccharide biosynthesis genes (genes 2050 to 2058), and genes encoding a homolog of MMO (PxmABC; genes 3483 to 3486) were downregulated under both nitrate and ammonium conditions, while the expression patterns between the *mbaI* mutant and the wild-type strain were reversed (Fig. 6; Table S5).

10.1128/mSystems.00770-19.9 TABLE S5 Normalized transcript counts for the MbaI mutant versus the TunJ mutant versus the wild type (experiment 4). Download Table S5, XLSX file, 0.8 MB. Copyright © 2020 Yu et al. 2020 Yu et al. This content is distributed under the terms of the Creative Commons Attribution 4.0 International license.

## DISCUSSION

In this study, we initiated the quest for untangling the complex mechanistic details of the hypoxia response employed by species of the *Methylobacter* genus that are aerobic methanotrophs. This stress response system uniquely combines modules of central carbon metabolism (i.e., methanol oxidation), denitrification, quorum sensing, and a secondary metabolite, tundrenone. This complex system may be responsible for the persistence and activity of *Methylobacter* species across gradients of dioxygen tensions and for their cosmopolitan distribution in freshwater and soil environments in the Northern Hemisphere, including the fast-melting permafrosts. One of the key elements of this hypoxia response system is the Hcp/Hcr pair of proteins that serve as a NO reductase. While Hcp and Hcr are rather widespread among *Proteobacteria* inhabiting both oxic and anoxic environments (31), to our knowledge, *Methylobacter* presents a unique case in which transcription from these genes appears to be controlled by a homolog of NorR that has been previously demonstrated to directly sense NO (32). Thus, NO appears to act as one of the signaling molecules in the hypoxia response. A hemerythrin (Hmr) encoded by the same gene cluster likely binds O_2_, as has been demonstrated for a homolog from a related *Methylococcales* species, Methylococcus capsulatus Bath (33). Thus, O_2_ appears to be another signaling molecule that Hmr potentially transfers to the PAS domain of the signal transduction protein named Hst here, while this domain could potentially also sense NO (34). The presence of the EAL and GGDEF domains (35) in the Hst protein provides support for the existence of a downstream regulatory cascade mediated by cyclic diguanylate, thus implying further complexity of the hypoxia response switch. Complex regulatory patterns have been previously proposed for some of the methylotrophy functions, such as the methanol oxidation functions, involving multiple transcriptional regulators (36, 37). From the data presented here, it appears that methanol oxidation is also regulated at the transcriptional level as part of the hypoxia response cascade, potentially responding to NO, O_2_, and additional regulators such as NorR.

Importantly, while the traditional respiratory denitrification functions (Nar and Nir) were expressed under both oxic and hypoxic conditions, their transcription patterns did not follow the ones of the *hcp*-*hcr* genes. One intriguing scenario that emerges from these data is that the main role of the respiratory denitrification pathway may be in producing the signaling molecule NO in response to hypoxia rather than in serving as a major energy-generating pathway.

**FIG 7:**
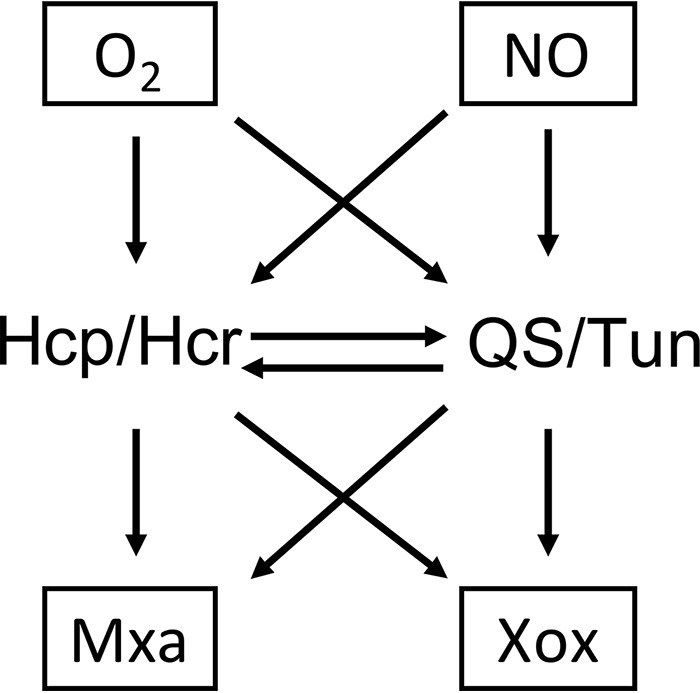
Simplified schematic of the complex interconnections between O_2_ and NO, likely directly sensed by regulatory and signal-transducing proteins, Hcp/Hcr and QS/Tun systems, and their effect on each other and on some key methylotrophy functions.

The functional interconnection between the Hcp/Hcr and the QS/Tun systems presents the next level of complexity and suggests a potential role for the recently discovered Tun as a novel signaling molecule whose synthesis, in turn, involves a more traditional signal transducer, HSL (Fig. 7). It is also possible that the QS/Tun system may respond to NO directly, as has been previously described for other QS systems (38, 39).

Why would such complexity be necessary? It is tempting to speculate that the complex interplay between different signals and regulators and their further interconnection with central metabolic pathways constitute a very robust system that allows *Methylobacter* to better adapt to changing environmental factors such as fluctuations in dioxygen availability and, potentially, to combinations of stressors. This hypothesis would be consistent with the persistence of *Methylobacter* over other aerobic methanotrophs, including the closely related genera *Methylosarcina* and *Methylomonas*, in methane-fed laboratory microcosms, especially under hypoxic conditions (13, 14). While carbon metabolism schemes between representatives of *Methylococcaceae* are very similar (6), they appear to be differentially equipped for stress responses. While *Methylosarcina* and *Methylomonas* may or may not encode the Hcp/Hcr pair and other respiratory denitrification enzymes, none of their known genomes encode the QS/Tun module (6). At the same time, it has been demonstrated that signaling molecules produced by *Methylobacter* may elicit a response by members of the natural community inhabiting the same niche, such as *Methylomonas* (40). On the other hand, it has been demonstrated that *Methylobacter* may respond to signals from other bacteria by altering its methylotrophy scheme (16). Intriguingly, the QS/Tun module in *Methylobacter* appears to be encoded by a genomic island (29); thus, it can be potentially spread among the community members under specific selective pressures, such as prolonged hypoxia, or be lost without such selective pressures, for example, as a result of a drought.

The details and the role of the hypoxia response described here in the environmental fitness of *Methylobacter* need further investigations, via both broader analyses of environmental microbiomes as well as laboratory manipulation of methanotrophs differentially equipped for a hypoxia response. However, the importance of this response likely extends beyond single-organism physiology, as aerobic methanotrophy is increasingly recognized as a community function. Thus, perhaps, further advances into the hypoxia response should involve not only single-species-based experiments but also community-based analyses.

## MATERIALS AND METHODS

### Strains and growth conditions.

Methylobacter tundripaludum 21/22 and Methylobacter tundripaludum 31/32 (6) were routinely cultivated in nitrate mineral salts (NMS) medium (41) with 25% methane and 75% (vol/vol) air in the headspace, with shaking at 200 rpm at 18°C, or on NMS medium solidified with 1.5% agar (Difco, Thermo Fisher Scientific, Waltham, MA, USA) and placed under a methane-air atmosphere in tight jars. The headspace atmosphere was created essentially as previously described (13, 14, 16–19). For certain experiments, ammonium mineral salts (AMS) medium (41) was used as per the experimental design (see below). All the glassware employed was acid washed for 24 h in 1 M hydrochloric acid before use, to remove trace amounts of any metals that may be adhering to the glass, such as lanthanides, which are known to affect the expression of alternative methanol dehydrogenases (15, 16). For some experiments, the headspace atmosphere was altered by replacing dioxygen with dinitrogen in order to create hypoxic conditions as per the experimental design.

### Genetic manipulations.

All genetic manipulations were achieved through electroporation of the assembled PCR amplification-based constructs into the cells of M. tundripaludum 31/32, followed by the selection of chromosomal recombinants, as previously described (42). Briefly, for the gene knockouts, the kanamycin resistance gene cassette (amplified from plasmid pCM433 [43]) and the two regions flanking the gene of interest were assembled by fusion PCR (44). The assembled fragment was further amplified using nested primers at a high annealing temperature (44). The resulting product was purified by using the QIAquick PCR purification kit (Qiagen), and the product was directly electroporated into M. tundripaludum 31/32. After electroporation, cells were precultivated overnight in liquid NMS medium without kanamycin, after which cells were harvested and transferred onto either NMS or AMS plates containing 50 μg/ml kanamycin. Single colonies were selected, and the desired mutation was verified by sequencing of the respective PCR-amplified fragment of the chromosomal DNA.

### Measurement of growth rates.

To investigate the growth dynamics of mutant strains in comparison to wild-type M. tundripaludum 31/32, cultures were placed into 30-ml tubes sealed with rubber stoppers (Wheaton, Millville, NJ, USA). Each culture (10 ml) was incubated in either NMS or AMS medium with 25% methane in the headspace and various dioxygen concentrations. For “high-oxygen” conditions, a methane/air ratio of 25:75 was used. For “low-oxygen” conditions, a methane/air/dinitrogen ratio of 25:5:70 was used. To create the former conditions, tubes were flushed with air for 2 min to equalize pressure, 5 ml of air was then removed by using a syringe, and 5 ml of CH_4_ was injected into the headspace. To create the latter conditions, tubes were flushed with dinitrogen for 2 min to equalize pressure, 6 ml of the gas phase was then removed by using a syringe, and 5 ml of CH_4_ and 1 ml of air were injected into the headspace. Tubes were incubated with shaking (200 rpm) at 18°C. Optical density at 600 nm (OD_600_) measurements were carried out using a Jenway 7300 spectrophotometer (Bibby Scientific, Burlington, NJ, USA) every 4 h. All values presented with a ± sign represent a minimum of 3 measurements, with ± reporting the standard error.

### Live/dead cell tests.

To obtain real-time relative dead/live cell abundances, cell numbers were determined by flow cytometry after staining with the LIVE/DEAD BacLight bacterial viability kit (Thermo Scientific, Waltham, MA, USA), according to the manufacturer’s instructions. For the analysis, 10 μl of the cell sample was mixed with 10 μl of LIVE/DEAD dye (1:100 in dimethyl sulfoxide [DMSO]) and 0.22-μm filtered NMS medium to a final volume of 830 μl per sample. These samples were incubated for 15 min in the dark at room temperature. Cells were measured with a CyFlow Space flow cytometer (Partec, Münster, Germany) with the following parameters: a laser emitting at 488 nm, fluorescence collected in the green and red channels, side scatter, green fluorescence and red fluorescence analyzed and displayed in log_3_ or log_4_, flow rate kept between 4 and 6 μl/s, and particle analysis rate at below 1,000 particles/s.

### Stress response experiments.

To test the hypoxia response of the mutants, cells of the seven mutants and the wild-type control were grown in 30-ml tubes, as described above, in both NMS and AMS media, starting with cultures at an OD_600_ of approximately 0.4. The high-oxygen headspace was created once and not replenished for 40 days. Survival rates were determined using LIVE/DEAD stain. A similar, short-term experiment was run over 12 h, and samples were taken every 4 h. For oxidative and nitrosative stress responses, 0.1 mM H_2_O_2_, paraquat, or DETA-NO, all purchased from Sigma-Aldrich (St. Louis, MO, USA), was added to the cultures under a methane-air (25:75) atmosphere, and samples for dead/live tests were taken every 4 h. To select for the specific concentration of each stressor, series of preliminary experiments were carried out using plate assays with H_2_O_2_ and paraquat over 10 days, with concentrations ranging from 0.01 to 2 mM. Concentrations of 0.1 mM and above were sufficient to prevent the growth of each of the mutants, while the wild-type strain was able to grow. As DETA-NO is oxygen sensitive, only liquid culture experiments were carried out, with 0.02 and 0.1 mM DETA-NO. The latter concentration produced a stronger negative response by the mutants.

### Instantaneous dissolved gas detection.

Cells were grown under standard conditions in AMS medium to the mid-exponential stage (OD_600_ of approximately 0.8). Cells were then concentrated by centrifugation (4,500 × *g* for 10 min) and resuspended in NMS medium to an OD_600_ of approximately 1.5, and cell suspensions (40 ml) were immediately placed into a 60-ml triple-port microrespirometry (MR) chamber (Unisense, Aarhus, Denmark). All MR experiments were performed at room temperature (approximately 20°C). O_2_ dynamics were measured using a Stox oxygen microsensor (Unisense, Aarhus, Denmark). The sensor body was partially wrapped with parafilm to fit into the port on the MR chamber. N_2_O and NO concentrations were measured using an N_2_O-MR sensor (Unisense) and a NO-MR sensor (Unisense), respectively. For O_2_ and N_2_O sensors, calibration was conducted according to the manufacturer’s instructions. For the NO sensor, 100 mM freshly prepared *S*-nitroso-*N*-acetyl-d,l-penicillamine (SNAP; Cayman Chemical, MI, USA) in a 0.1 M CuCl_2_ solution was used for calibration. All sensors were plugged directly into a microsensor multimeter (Unisense) and polarized for >1 day prior to use. All data were logged on a laptop via the microsensor multimeter using SensorTrace Logger software (Unisense).

### Gene expression analysis.

**(i) Experiment 1: time-resolved hypoxia response.** Experiment 1 was described previously (17). Briefly, synthetic communities were assembled by mixing 4 strains (experiment 1A) or 14 strains (experiment 1B). In the 4-strain community, M. tundripaludum 31/32 was the only methanotroph species, and in the 14-strain community, M. tundripaludum 31/32 was one of the two methanotroph species. The communities were grown in NMS medium with 25%:75% (vol/vol) methane-air in the headspace. After 24 h and at an OD_600_ of approximately 0.5, samples were flushed with the same atmosphere, and replicate samples were sacrificed after 1, 24, and 48 h, with the first sample being oxic and the latter samples becoming increasingly hypoxic.

**(ii) Experiment 2: Hcp mutant versus the wild type.** Hcp mutant and wild-type cells were grown under high-oxygen conditions in either NMS or AMS medium to an OD_600_ of approximately 0.5; these cells were then used to inoculate replicate cultures, and these cultures were incubated as described above, for another 24 h, to an OD_600_ of approximately 0.5. At this point, half of the samples were given the same methane-air atmosphere for 1 h, after which these (oxic) samples were collected. The second set of samples was given an atmosphere of methane-dinitrogen (25%:75%, vol/vol) to create hypoxic conditions, and the samples were collected after 24 h.

**(iii) Experiment 3: NasA mutant NO challenge.** The NasA mutant was grown under high-oxygen conditions in AMS medium to an OD_600_ of approximately 0.5. At this point, half of the samples were pelleted by centrifugation (4,500 × *g* for 10 min) and reinoculated into AMS medium. The second set of samples was pelleted by centrifugation and reinoculated into NMS medium. All the samples were incubated under high-oxygen conditions for 1 h. After incubation, the cells were centrifuged and used for RNA extraction.

**(iv) Experiment 4: MbaI and TunJ mutants versus the wild type.** MbaI and TunJ mutants and wild-type M. tundripaludum 21/22 were grown in either NMS or AMS medium without replenishing the headspace atmosphere (25%:75% methane-air). Cells were harvested for RNA isolation after 48 h.

RNA was extracted as described previously (17, 19). cDNA library preparation and RNA sequencing were performed by Genewiz (South Plainfield, NJ, USA), using Illumina HiSeq 2-by-150 (paired-end) reads. The raw reads from the sequencing facility were aligned to the corresponding genome assemblies (6), both of which were downloaded from the Joint Genome Institute IMG website (https://img.jgi.doe.gov/). Alignments were performed using BWA version 0.7.12-r1044 using the BWA-MEM algorithm and default parameters (45). The alignments were postprocessed into sorted BAM files with SAMtools version 1.2-232-g87cdc4a (46). Reads were attributed to open reading frames (ORFs) using the htseq-count tool from “HTSeq” framework version 0.6.1p1 in the “intersection-nonempty” mode (47). Differential abundance analysis was performed with DESeq2 1.2.10 (48) using R 3.3.1.

### Data availability.

Transcriptomics data have been deposited under BioProject accession number PRJNA575263.
