# Supplementary figures and images for: A Complex Interplay between Nitric Oxide, Quorum Sensing, and the Unique Secondary Metabolite Tundrenone Constitutes the Hypoxia Response in Methylobacter
Source: mSystems. 2020 Jan 21;5(1):e00770-19. doi: 10.1128/mSystems.00770-19 (PMC6977074; doi:10.1128/mSystems.00770-19)

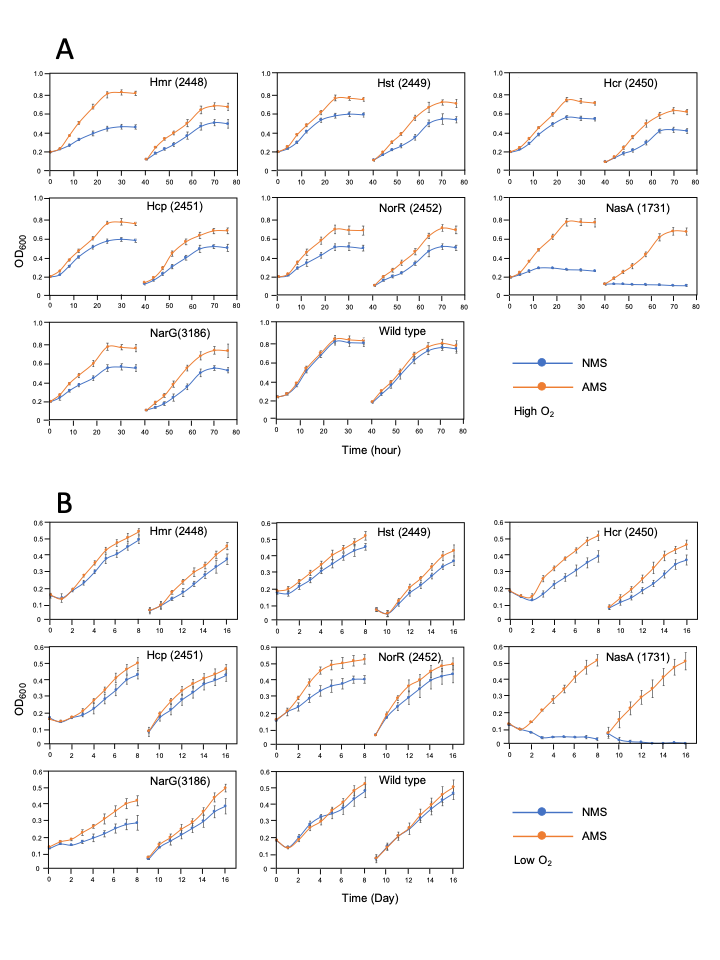

Supplement: FIG S1 [file mSystems.00770-19-sf001.tif]

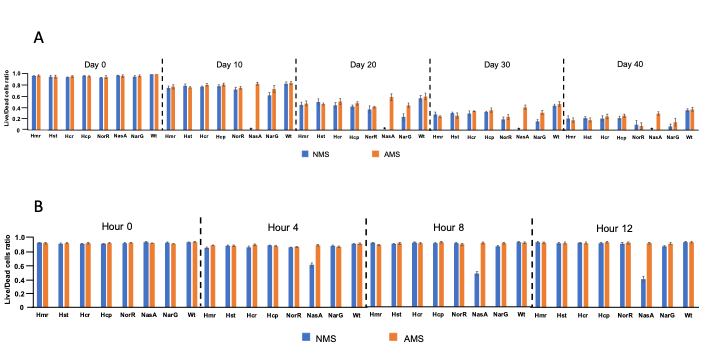

Supplement: FIG S2 [file mSystems.00770-19-sf002.tif]

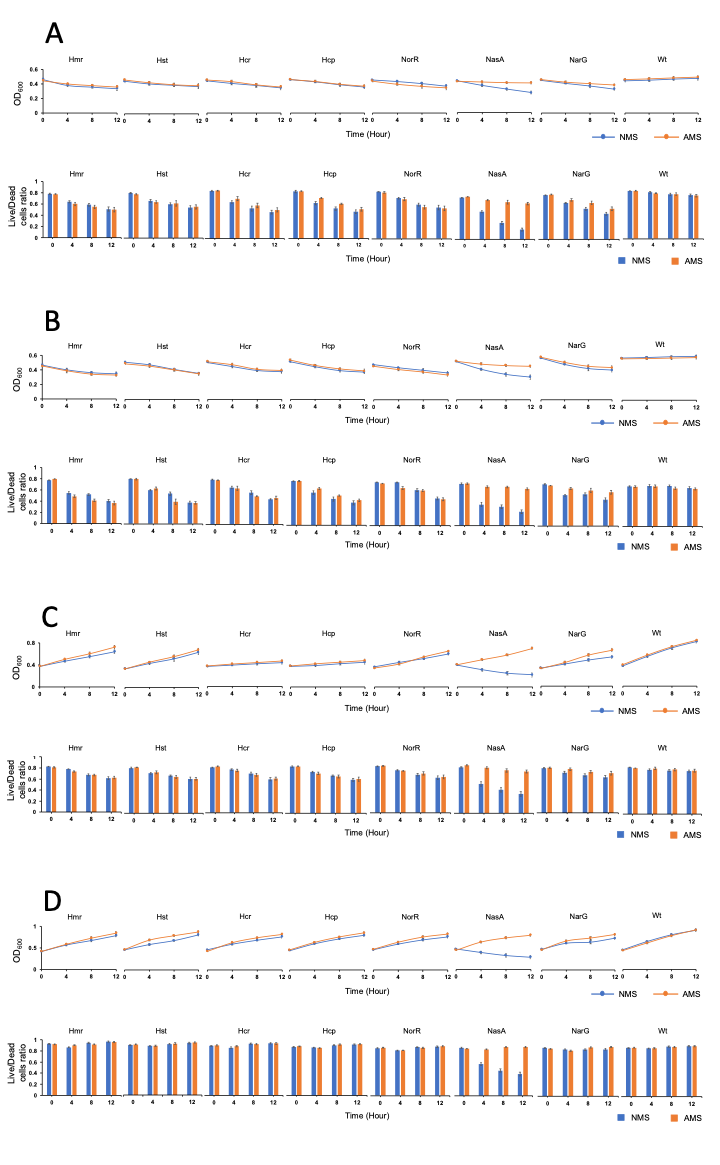

Supplement: FIG S3 [file mSystems.00770-19-sf003.tif]

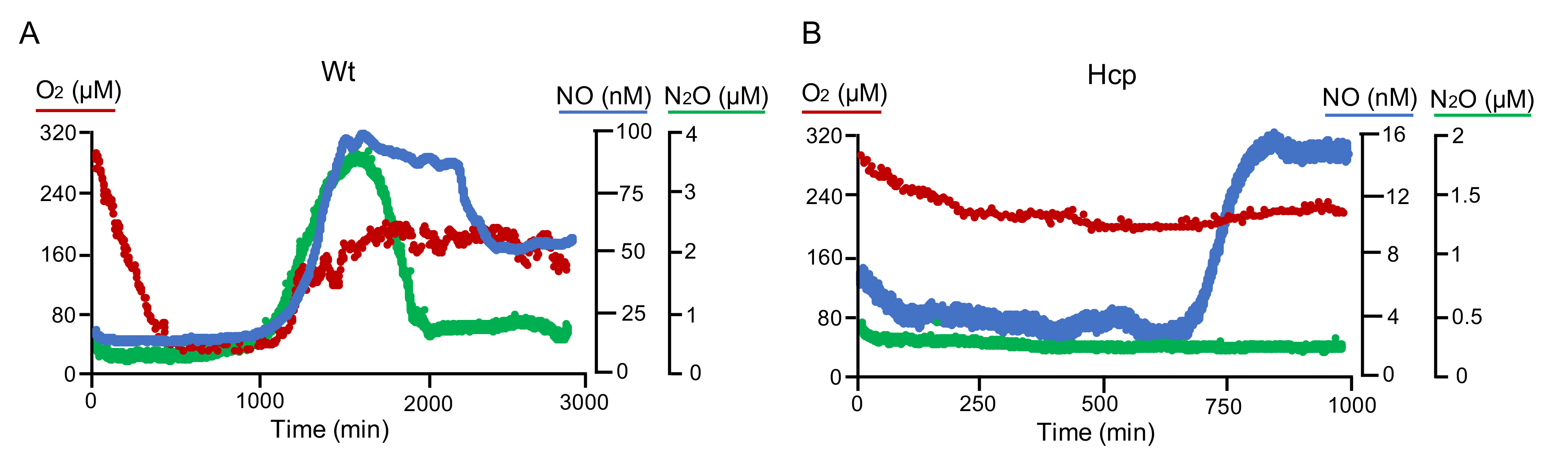

Supplement: FIG S4 [file mSystems.00770-19-sf004.tif]
